# The association between the body height and cardiovascular diseases: a retrospective analysis of 657,310 outpatients in Germany

**DOI:** 10.1186/s40001-022-00881-y

**Published:** 2022-11-09

**Authors:** Sarah Krieg, Karel Kostev, Mark Luedde, Andreas Krieg, Tom Luedde, Christoph Roderburg, Sven H. Loosen

**Affiliations:** 1 Clinic for Gastroenterology, Hepatology and Infectious Diseases, University Hospital Düsseldorf, Medical Faculty, Heinrich Heine University Düsseldorf, Moorenstrasse 5, 40225 Düsseldorf, Germany; 2 IQVIA, Frankfurt, Germany; 3 KGP Bremerhaven, Bremerhaven, Germany; 4 Department of Surgery (A), University Hospital Düsseldorf, Medical Faculty Heinrich Heine University Düsseldorf, Moorenstrasse 5, 40225 Düsseldorf, Germany

**Keywords:** Arterial hypertension, Atrial fibrillation, Myocardial infarction, Cardiovascular disease, Prevention

## Abstract

**Background:**

Cardiovascular disease (CVD) represents the leading cause of death worldwide. The identification of individuals at increased risk of CVD is essential to reduce its morbidity and mortality globally. Based on existing data on a potential association between the individual body height and the risk for CVD, we investigated this association in a large cohort of outpatients in Germany.

**Methods:**

A total of 657,310 adult outpatients with available body height data from the Disease Analyzer (IQVIA) database were included in Germany between 2019 and 2021. The prevalence of common CVD diagnoses (hypertension, coronary heart disease, atrial fibrillation and flutter, heart failure, ischemic stroke, and venous thromboembolism) was evaluated as a function of the patients’ body height stratified by age and sex.

**Results:**

In both sexes, the prevalence of hypertension, coronary heart disease, heart failure, and ischemic stroke was higher among patients of smaller body height. In contrast, the prevalence of atrial fibrillation and venous thromboembolism was higher in taller patients. In age- and BMI-adjusted logistic regression analyses, an increased body height was negatively associated with coronary heart disease (OR = 0.91 in women and OR = 0.87 in men per 10-cm increase in height) and strongly positively associated with atrial fibrillation (OR = 1.25 in women and men) and venous thromboembolism (OR = 1.23 in women and OR = 1.24 in men).

**Conclusion:**

We present the first data from a large cohort of outpatients in Germany providing strong evidence for an association between the body height and common CVD. These data should stimulate a discussion as to how far the body height should be implemented as a parameter in stratification tools to assess CVD risk in order to further reduce cardiovascular morbidity and mortality in the future.

## Introduction

Cardiovascular disease (CVD) is the leading cause of death worldwide and a major contributor to disability [1]. In Europe, more than 4 million people die of CVD each year, of whom 1.4 million are younger than 75 years [2, 3]. Besides well known risk factors, such as age, gender, genetic predisposition, hypertension, diet, dyslipidemia, diabetes, obesity, smoking, physical inactivity, and psychosocial factors [4–6], body height has recently been linked to the occurrence of various CVD in different populations [7–9]. The Emerging Risk Factors Collaboration (ERFC) recently showed that the risk of dying from coronary heart disease (CHD) decreased by 6% for every 6.5 cm of adult body height in a cohort of over 1 million participants [7]. Similar results were provided by a meta-analysis of Mendelian randomization that included individual participant data from 60,028 participants of European ancestry from 21 prospective studies [10]. In this regard, shorter body height has also been linked to risk factors for developing CHD, including hypertension, high levels of low-density lipoprotein (LDL) cholesterol, and diabetes [10]. In contrast, a positive association between an increasing body height in adulthood is reported with the risk of developing atrial fibrillation (AF) [10, 11]. Adult body height can be considered as a marker reflecting an interplay of genetic predisposition and various earlier life circumstances, such as nutritional, social, and psychological [8, 12]. Over the years, an increase in adult body height has been observed in industrialized countries, which may be due to improved nutrition, higher socioeconomic status, and lower prevalence of diseases than in poor countries [8, 12]. To reduce cardiovascular morbidity and mortality, prevention strategies need to be developed that identify vulnerable risk groups in different populations [13]. Due to their simplicity and noninvasiveness, anthropometric measures are very attractive indices for assessing a person’s susceptibility to disease [14]. For the estimation of individual CVD risk, professional societies, such as the European Society of Cardiology (ESC), recommend prognostic models that incorporate multiple risk factors using risk algorithms, with body height previously considered only in the form of body mass index (BMI) [15–17]. The purpose of this study was to investigate the association between body height and CVD risk in a large cohort of over 650,000 outpatients in Germany.

## Materials and methods

### Database

This study used data from the Disease Analyzer database (IQVIA), which has been extensively described in the literature [18]. The Disease Analyzer database contains demographic, diagnosis, and prescription data from patients followed in general and specialized practices in Germany. Practices to include in the database are selected based on multiple factors (i.e., physician’s age, specialty group, community size category, and German federal state), and the database is composed of around 3–5% of all practices in Germany. Diagnosis and prescription data are coded using the International Classification of Diseases, 10th revision (ICD-10), and the Anatomical Classification of Pharmaceutical Products of the European Pharmaceutical Marketing Research Association (EphMRA), respectively. Finally, data are anonymously sent to IQVIA on a regular basis, and the quality of these data is assessed using several criteria, such as completeness of documentation and linkage between diagnoses and prescriptions.

### Study population

This retrospective cohort study included 657,310 individuals followed in one of 757 GP in Germany between January 2019 and December 2021. The only inclusion criteria was at least one documented body height value. As body height usually does not change over time, there was no special time period for body height documentation for these patients; body height value has to be available at least once between January 2014 and December 2021. Body height values were available for 657,310 (14.2%) out of 4,619,156 individuals followed in the 757 practices.

### Study outcomes and variables

The outcome of this study was the prevalence of pre-defined diagnoses in the study time period as a function of body height. Body height was included in this study as a four-category variable: for women ≤ 160 cm, 161–170 cm, 171–180 cm,  > 180 cm and for men ≤ 165 cm, 166–175 cm, 176–185 cm,  > 185 cm. Diagnoses included hypertension (ICD-10: I10), coronary heart disease (ICD-10: I24, I25), atrial fibrillation and flutter (ICD-10: I48), heart failure (ICD-10: I50), ischemic stroke (ICD-10: I63, I64), and venous thromboembolism (ICD-10: I80).

### Statistical analyses

Age at first visit in 2019–2021 was compared between body height categories. As there was a strong relationship between body height and age (higher people was younger), all analyses were performed either by age group or adjusted for age. First, 3-year prevalence of study diseases by age group and sex was descriptively shown. Then, associations between body height and these diseases by sex were analyzed with logistic regression models with diagnoses as dependent variables and height (per 10-cm increase in height) as impact variables adjusted for age and body mass index. The results of the regression analyses are displayed as odds ratios (ORs) and 95% confidence intervals (95% CI) for each diagnosis. Due to multiple comparison and high patient samples, *P*-values lower than 0.001 were considered statistically significant. Analyses were conducted with SAS 9.4 (SAS Institute, Cary, US).

## Results

### Patient characteristics

This study included a total of 348,478 female and 308,832 male patients. The mean age (SD) was 51.7 years (17.4 years) among women and 50.7 years (16.8 years) among men, respectively. In women, the average body height was 164.8 cm and the average body mass index (BMI) was 27.9 kg/m^2^. Men showed an average body height and BMI of 177.9 cm and 27.9 kg/m^2^ (Table 1).

**Table 1:** Age and body height of study patients

Variable	Women				
	Total	≤ 160 cm	161–170 cm	171–180 cm	> 180 cm
N	348,478	100,583	184,645	59,784	3466
Age (mean, SD)	51.7 (17.4)	54.4 (17.8)	51.0 (17.0)	45.2 (15.3)	41.0 (13.3)
Height (mean, SD)	164.8 (6.8)	156.8 (3.3)	165.8 (2.7)	174.2 (2.6)	183.7 (2.7)
BMI (mean, SD)	27.0 (7.0)	27.9 (6.4)	26.8 (7.5)	26.1 (6.1)	25.9 (6.2)
	Men				
Variable	Total	≤ 165 cm	166–175 cm	176–185 cm	> 185 cm
N	308,832	14,816	102,088	144,359	47,569
Age (mean, SD)	50.7 (16.8)	59.4 (18.0)	54.6 (17.1)	49.1 (17.1)	44.1 (14.5)
Height (mean, SD)	177.9 (7.4)	162.4 (3.1)	171.7 (2.6)	180.1 (2.8)	189.5 (3.4)
BMI (mean, SD)	27.9 (5.1)	28.2 (5.4)	28.1 (5.1)	27.8 (5.1)	27.6 (5.2)

### Association between body height and cardiovascular disease

For both, women and men, the prevalence of hypertension, CHD, heart failure, and ischemic stroke stepwise decreased with higher body height (Table 2). This finding was consistent for almost all analyzed age groups (Table 2). In contrast, the prevalence of AF and venous thromboembolism (VTE) stepwise increased with a higher body height (Table 2).

**Table 2:** Prevalence of cardiovascular diseases by age and body height categories

Age group	Women				Men			
	≤ 160 cm	161–170 cm	171–180 cm	> 180 cm	≤ 160 cm	161–170 cm	171–180 cm	> 180 cm
Hypertension								
≤ 50 years	18.1	16.2	14.2	14.6	22.1	22.8	21.5	20.7
51–60 years	45.0	39.3	35.1	31.4	48.1	47.8	44.8	41.9
61–70 years	55.5	52.3	50.5	38.6	56.8	55.9	54.2	50.6
> 70 years	59.3	59.6	56.7	62.0	55.2	57.0	55.8	54.0
Coronary heart disease								
≤ 50 years	1.4	1.1	0.9	0.7	4.7	3.8	2.9	2.2
51–60 years	6.3	4.7	3.4	3.3	15.0	14.3	11.5	8.9
61–70 years	11.4	9.6	8.8	8.2	24.1	21.5	18.1	15.9
> 70 years	16.8	15.5	14.0	13.9	27.8	26.1	24.2	21.6
Atrial fibrillation and flutter								
≤ 50 years	0.4	0.5	0.7	0.7	1.1	1.1	1.2	1.6
51–60 years	2.2	2.7	3.2	3.8	3.8	4.3	5.1	5.7
61–70 years	6.8	7.8	10.2	9.9	8.7	10.2	12.3	13.8
> 70 years	14.9	16.7	19.2	16.5	15.8	18.5	20.9	24.4
Heart failure								
≤ 50 years	0.8	0.7	0.6	0.6	1.6	1.2	1.2	1.1
51–60 years	3.6	3.1	2.5	2.8	6.1	5.2	4.5	4.3
61–70 years	8.4	7.0	6.6	4.4	12.3	9.8	9.0	9.4
> 70 years	17.1	15.3	15.0	16.5	18.7	17.4	16.3	16.2
Ischemic stroke								
≤ 50 years	0.4	0.3	0.3	0.4	0.9	0.7	0.5	0.4
51–60 years	1.3	1.2	1.0	1.1	3.2	2.6	2.1	1.9
61–70 years	2.6	2.4	2.3	2.5	4.6	4.4	3.4	3.0
> 70 years	4.7	4.2	4.2	5.1	6.3	5.7	5.1	4.7
Venous thromboembolism								
≤ 50 years	0.8	1.1	1.2	1.7	0.5	0.8	0.9	1.1
51–60 years	1.6	1.8	2.3	2.3	1.3	1.5	1.7	2.5
61–70 years	2.3	2.7	3.3	1.3	2.0	2.1	2.4	3.2
> 70 years	3.1	3.5	4.1	5.1	2.3	2.6	2.6	3.7

Results were confirmed in age- and BMI-adjusted logistic regression models (Table 3). Here, a taller body height (effect per 10 cm of body height increase) was negatively associated with CHD (OR = 0.91, 95% CI 0.89–0.93, *P* < 0.001 in women and OR = 0.87, 95% CI 0.85–0.88, *P* < 0.001 in men). Moreover, there was a negative association between body height and hypertension in women (OR = 0.97, 95% CI 0.96–0.99, *P* < 0.001) as well as between body height and ischemic stroke (OR = 0.90, 95% CI 0.86–0.92, *P* < 0.001) in men. Vice versa, a taller body height was positively associated with AF (OR = 1.25, 95% CI 1.22–1.28, *P* < 0.001 in women and OR = 1.25, 95% CI 1.23–1.28, *P* < 0.001 in men) and VTE (OR = 1.23, 95% CI 1.18–1.27, *P* < 0.001 in women and OR = 1.24, 95% CI 1.19–1.29, *P* < 0.001 in men, Table 3).

**Table 3:** Association between body height and cardiovascular diagnoses

Disease	Women		Men	
	OR (95% CI) per 10-cm increase in height*	*P*-values	OR (95% CI) per 10-cm increase in height*	*P*-values
Hypertension	0.97 (0.96–0.99)	< 0.001	0.99 (0.98–1.01)	0.116
Coronary heart disease	0.91 (0.89–0.93)	< 0.001	0.87 (0.85–0.88)	< 0.001
Atrial fibrillation and flutter	1.25 (1.22–1.28)	< 0.001	1.25 (1.23–1.28)	< 0.001
Heart failure	0.98 (0.96–1.00)	0.098	0.98 (0.96–1.00)	0.039
Ischemic stroke	0.94 (0.91–0.98)	0.002	0.90 (0.86–0.92)	< 0.001
Venous thromboembolism	1.23 (1.18–1.27)	< 0.001	1.24 (1.19–1.29)	< 0.001

^*^Multivariable logistic regression adjusted for age and body mass index

## Discussion

In this study, we evaluated the association between the body height and common CVD stratified by sex and age group in a large cohort of over 650,000 outpatients in Germany. In line with previous studies [7–9], we found evidence that the individual body height is distinctly associated with the risk of developing CVD. Age- and BMI-adjusted logistic regression analysis indicated that an increase in body height is negatively associated with CHD but positively associated with AF as well as VTE. This association was found in both sexes.

A potential association between the body height and CVD has been previously evaluated outside from Germany. Nelson et al. analyzed genotype data from 18,249 individuals in relation to CHD risk using a genetic approach [8], finding an association between a genetic decrease in body height and increased CHD risk [8]. The authors identified overlapping pathways that link height-associated single nucleotide polymorphisms (SNPs) potentially influencing CAD risk. Among these, the bone morphogenetic protein (BMP) and transforming growth factor beta (TGF-β) signaling pathways, the axon guidance pathway, and the signal transducer and activator of transcription 3 (STAT3) and insulin-like growth factor-1 (IGF-1) pathway are reported to simultaneously play experimentally documented roles in the development of atherosclerosis. Overlapping and complex biological pathways are therefore thought to influence body height, as well as atherosclerosis risk, through effects on vascular biology and function. Interestingly, the authors also found a significant overall association between height-related SNPs and LDL cholesterol and triglycerides [8]. Although the mechanisms by which SNPs associated with body height might affect LDL cholesterol and triglyceride levels are not yet known, the authors suggested that these effects in combination might partially explain the link between genetically determined shorter body height and increased CHD risk [8]. Furthermore, body height is reported to correlate positively with coronary artery diameter, suggesting that this would be a hypothetical simple explanation for increased CHD risk in smaller individuals. Accordingly, smaller individuals have proportionally smaller coronary arteries, so a similar plaque burden could lead to a greater likelihood of symptomatic disease [8, 19].

Our observation that an increasing body height, to the contrary, has a positive association with AF also has been noted in previous studies in other populations [20–22]. In a large Korean cohort study, data from the Korean National Health Insurance Service-National Sample Cohort (NHIS-NSC) from 2002 to 2015 of more than 300,000 individuals who underwent a medical examination between 2006 and 2009 were analyzed. Performing multivariate statistical analysis, the authors found that after adjusting the data for various confounders, a 5-cm increase in body height raised the risk of AF by 1.22 times [23]. Schmidt et al. conducted a 36-year cohort study of men born between 1955 and 1965 [24]. For this purpose, data from the Danish National Patient Register were analyzed relating body height to several variables, including AF. As a result, they found that tall people had a higher risk of AF when compared to people of smaller stature. Similarly, the Copenhagen City Heart Study analyzed 18,852 randomly selected men and women aged 20–93 years who did not have AF at baseline in four cross-sectional studies between 1976 and 2003. This showed that the risk of AF increased by 35–65% for every 10-cm difference in body height in both men and women [25]. Rosenberg MA et al. analyzing a large cohort of older adults demonstrated that greater body height was associated with the incidence and prevalence of AF, both after adjusting for confounders of body height and AF and after adjusting for other risk factors for AF. Accordingly, the risk of AF was predicted to increase by 35–65% with each 10-cm difference in body height [20]. AF is known to be a multifaceted disease process determined by structural, neural, electrical, and hemodynamic factors. Increased atrial size is considered a risk factor for developing AF [26] and numerous studies have already reported a positive correlation between enlarged atria and AF [27–29]. Studies suggest a direct correlation of body height with atrial and ventricles size. As a result of enlargement of the atria, increasing body height might lead to abnormal conduction patterns, autonomic dysregulation, and, correspondingly, the development of AF. Interestingly, the size effect has also been noted in various experimental and observational studies in animals [30, 31]. For example, a higher prevalence of AF was found in larger animals, such as horses compared with smaller animals, whereas it seems to be impossible to induce AF in mice [30, 31]. A possible cause has been discussed to be the smaller size of the left atrium (LA) in smaller animals [31]. Given the constantly increasing body height in developed countries, the link and underlying mechanisms should be better understood and screening parameters for taller people should be considered to prevent and treat AF and its complications prior to their occurrence.

In addition, our study also showed a strong association between the body height and VTE, which is consistent with the results of other epidemiological studies [32]. For example, in a large study using Swedish national registry databases, this association was demonstrated by the use of a co-sibling design aimed at reducing the influence of family confounders. Compared with the tallest women (> 185 cm) and men (> 190 cm), a graded reduction in risk was found with lower body height in both sexes [32]. Similarly, a Mendelian randomization study indicated that increased body height is a positive predictor of VTE [33]. For the association between body height and VTE, it is hypothesized that taller people have increased hydrostatic pressure in the vessels due to longer extremities, which may result in greater stasis according to Virchow’s triad [34].

Some limitations of our study should be acknowledged. First, all diagnoses were documented with ICD-10 codes, which potentially leads to misclassification and undercoding of certain diagnoses. Our study examined the association between body height and various CVD or risk factors, such as hypertension, coronary artery disease, AF, heart failure, ischemic stroke, or VTE, which was adjusted for age, sex, and BMI. Information on socioeconomic status, environmental conditions, or lifestyle factors (e.g., nicotine and alcohol use) as well as data on mortality that would have allowed more detailed analyses were not available. Although the results of our study are supported by the results of previous prospective cohort studies, no causal relationships but only associations can be established. Nevertheless, the power of our study was the large number of patients enrolled and the use of representative data.

## Conclusions

We present the first data from a large cohort of patients from Germany that provide clear evidence of an association between body height and various CVD. For an optimized estimation of the individual overall risk for CVD, further studies are needed to clarify to what extent body height should be better considered as an independent risk factor in the future. Consideration should be given as to how far body height should be included as a separate screening parameter in risk stratification tools to detect cardiovascular risk constellations at an early stage and to further reduce cardiovascular morbidity and mortality.

## Data Availability

Data are available upon reasonable request from the corresponding author.
